# Mechanical dilatation of the stenosed cervix under local anesthesia: A prospective case series

**DOI:** 10.1111/jog.15179

**Published:** 2022-02-07

**Authors:** Kirsty V. Biggs, San Soo Hoo, Mallikarjun Kodampur

**Affiliations:** ^1^ Queen Mary University of London; ^2^ University Hospitals North Midlands NHS Trust

**Keywords:** anesthesia, cervical intraepithelial neoplasia, colposcopy, local, outpatients, uterine cervical neoplasms

## Abstract

**Aim:**

Cervical stenosis is traditionally managed by mechanical dilatation under general anesthesia (GA). We aimed to assess the safety, effectiveness, and patient acceptability of dilatation in the outpatient setting under local anesthesia (LA).

**Methods:**

Data were collected prospectively from all patients attending the outpatient department with cervical stenosis from March 20, 2015 to September 23, 2020. Mechanical dilatation of the cervix was performed using Hegar dilators under LA. Subsequent colposcopic assessment, cytology, histology, and management were recorded.

**Results:**

One hundred forty‐nine cases were referred for cervical dilatation, 63 (43%) of which had complete stenosis. One hundred eighteen (79%) patients had previously undergone cervical procedures. Successful dilatation under LA was achieved in 119 (83%) patients; 5 (3%) declined (requesting GA), 6 (4%) did not tolerate speculum examination, and 19 (13%) had unsuccessful procedures. The median Hegar size used was 8 mm. Dilatation under LA was acceptable in 93% attempted procedures. Thirteen episodes of restenosis were recorded with no major adverse events. Younger age (*p* = 0.045) and severe (compared to complete) stenosis (*p* < 0.0001) were associated with procedure success, with improved results over time (*p* = 0.003). Successful dilatation permitted cervical assessment; eight patients required cervical excisions, two underwent hysterectomies, with one confirmed case of adenocarcinoma.

**Conclusion:**

Rigid cervical dilatation in the outpatient setting provides effective, instantaneous treatment for women who have failed cytological or colposcopic assessment. For the vast majority of women, the procedure was well tolerated and preferred to using GA. However, given that 1 in 10 women experienced restenosis, patients should be counseled about the possibility of requiring further management.

## Introduction

Cervical stenosis has been defined as a cervical narrowing precluding the insertion of a 2.5 mm Hegar dilator.^1^, ^2^ The true incidence is unknown; however, a retrospective review of over 30 000 hysteroscopies identified cervical stenosis in a third of all cases.^3^ Several risk factors for the development of cervical stenosis have been identified, most frequently, large loop excision of the transformation zone (LLETZ) or conization with an incidence ranging from 1.3% to 16.8%.^2^, ^4^, ^5^, ^6^, ^7^, ^8^, ^9^ As a consequence, significant stenosis may result in the obstruction of menstrual flow, inadequate cervical assessment during colposcopy, unsuccessful gynecological procedures and potential implications with fertility.^1^, ^4^, ^10^, ^11^ Furthermore cervical stenosis has been reported, alongside pain, as the most frequent reason for failed hysteroscopy.^3^

Two main methods of cervical dilatation have been described in the literature, especially in the context of labor.^12^, ^13^, ^14^ Mechanical methods which include traditional dilators (such as Hern, Hegar, Pratt, Hanks, and Denniston dilators),^13^, ^15^ balloon catheters or osmotic dilators,^13^, ^16^ or pharmacological methods, namely prostaglandins or hyoscine butylbromide.^17^, ^18^, ^19^

Management of cervical stenosis using traditional dilators in the theater setting has been described in the literature.^20^, ^21^ Mechanical dilatation is often required prior to operative hysteroscopies, with crucial navigation of the internal os to allow successful insertion of surgical instruments.^11^ Mathew and Mohan, however recognized outpatient cervical dilatation as the first line management for cervical stenosis, with general or regional anesthesia reserved for those who cannot tolerate under a local anesthetic.^22^ In a retrospective study of over 10 000 women undergoing outpatient hysteroscopies, stenosis of the cervix was managed successfully with minimal discomfort in 98.5% of patients.^3^

In our unit, patients with cervical stenosis are offered mechanical dilatation under local anesthesia (LA). We conducted a prospective case series over a 5‐year period to assess the safety, effectiveness, and patient acceptability of using Hegar dilators in the outpatient setting.

## Methods

Data were collected prospectively from all patients attending the gynecology treatment suite with cervical stenosis from March 20, 2015 to September 23, 2020. Cervical stenosis was categorized as either significant (scarring with 2–3 mm opening of cervical os) or complete (scarring of the cervix with no os detected). All patients were referred following failed smear or colposcopy examination.

Patients have given written consent for the data collection. Personal information was collected at the clinic appointment including age, parity, number of previous LLETZ, contraceptive use, gynecological, and past medical history.

Mechanical dilatation of the cervix was performed using Hegar dilators under local anesthetic by a single operator. Mepivacaine hydrochloride 3% without adrenaline was administered as a paracervical block at 3 and 9 o'clock position including cervical stromal infiltration of LA at 12 and 6 o'clock position. The position of a stenosed cervical os was determined by a combination of digital and speculum examination to help identify the location of cervix and hence the potential cervical os position. Commonly, there was visible scarring or a dimple over the stenosed os. The scar was opened by sharp dissection either using a size 15 or 11 blade or McIndoes scissors or an artery forceps, following LA. This process allowed further dilatation with the insertion of Hegar dilator.

The size of Hegar dilator used was recorded, alongside any immediate complications. All women were enquired about their experience of undergoing the procedure under LA and if their preference to have the procedure under general anesthesia (GA).

The outcome following procedure including colposcopic assessment, cytology, histology and any further investigations were recorded were retrospectively collected through hospital clinical notes and letter. The results from all successive colposcopy and smear appointments were followed‐up until May 27, 2021. Data were also collected on any reported complications, including restenosis. In the event of an unsuccessful procedure, subsequent management decisions were recorded.

Microsoft Excel was used for the collation of basic percentage data. IBM SPSS 26 statistical software was used for all other statistical analysis. For categorical data Pearson's chi‐squared test was used to determine significant differences between groups. Fisher's exact test was used if cells had an expected count of less than 5. For nonparametric data, the Mann–Whitney *U* test was used. *p*‐Values < 0.05 were considered significant. Histograms were used to determine the data distribution.

## Results

### Patient characteristics

A total of 149 referrals were made for 148 patients to attend the gynecology treatment suite for mechanical dilatation of the cervix. Patient characteristics are described in Table 1. The patients' age ranged between 25 and 69 year with a median age of 50 years (39, 55). At the time of procedure, 78 patients (53%) were premenopausal, 6 (4%) were perimenopausal, and 64 (43%) were postmenopausal. Forty‐one patients were using contraceptive; including the combined oral contraceptive pill, progestogen only pill, Implanon, Mirena IUS, Depo‐provera, and condoms. Seven (5%) patients had been sterilized and in three (2%) cases, their partners had undergone vasectomy. Seventy‐nine percent of all patients had previous LLETZ procedures; 87 (59%) patients had undergone one LLETZ, 27 (18%) had had two LLETZ procedures, and four (3%) had undergone three LLETZ.

**TABLE 1:** Patient characteristics

Age	50 (39, 55)
Parity	2 (0, 3)
Contraceptive method	
Menopausal	64 (43)
Perimenopausal	6 (4)
POP	13 (9)
Implant	9 (6)
COCP	8 (5)
Mirena IUS	4 (3)
Depo provera	3 (2)
Sterilization	7 (5)
Vasectomy	3 (2)
Condoms	4 (3)
No contraceptive	27 (18)
Previous LLETZ	118 (79)
1 LLETZ	87 (59)
2 LLETZ	27 (18)
3 LLETZ	4 (3)
Previous O/G procedures	28 (19)
Presence of comorbidities	46 (31)

*Note*: All data is displayed as number (%) or median (interquartile range). Previous gynecological procedures: cesarean section (*n* = 15), sterilization (*n* = 7), cervical polypectomy (*n* = 3), endometrial ablation (*n* = 3), cervical dilatation (*n* = 2), evacuation of retained products of conception (*n* = 2), salpingo‐oophorectomy (*n* = 2), laparotomy (*n* = 1), dilation and curettage (*n* = 1), oophorectomy (*n* = 1), Comorbidities: hypertension (*n* = 12), cardiac other (*n* = 5), respiratory (*n* = 11), diabetes/endocrine (*n* = 10), musculoskeletal (*n* = 5), breast cancer (*n* = 3), gastrointestinal (*n* = 3), gynecological (*n* = 2), psychiatric (*n* = 2), Meniere's disease (*n* = 1), HIV (*n* = 1), Di‐George syndrome (*n* = 1).

Abbreviations: COCP, combined oral contraceptive pill; IUS, intrauterine system; LLETZ, large loop excision of the transformation zone; POP, progestogen only pill.

### Cervical dilatation

Cervical stenosis was assessed as severe in 75 (51%) of patients, and complete in 63 (43%). For the remaining 10 cases (7%), 5 patients had declined cervical dilatation, 2 patients did not tolerate the procedure, and 3 patients did not have data (Table 2). One patient had hematometra identified but subsequent pelvic ultrasound assessment was unremarkable.

**TABLE 2:** Cervical stenosis and dilatation

	*n* (%)/ median (IQR)
Degree of stenosis	
Severe stenosis	75 (51)
Complete stenosis	63 (43)
Not assessed/ missing data	10 (7)
Hematometra	1 (<1%)
Cervical dilatation	
Opted for GA without trial of LA (patient choice)	5/149 (3)
Procedure performed successfully under LA	119/144 (83)
Unable to tolerate procedure/examination	6/144 (4)
Failed procedure	19/144 (13)
Hegar dilator used (mm)	8 (7,8)
Patient preferred LA to GA (asked postprocedure)	128/137 (93)^a^
Complications	
Vasovagal episode	1/144 (<1)
Infection	1/144 (<1)
Restenosis episodes	13/119 (11)
Restenosis management	
Hysterectomy	8/13 (62)
Repeat dilatation under GA	2/13 (15)
Repeat dilatation under LA	2/13 (15)
LLETZ (GA)	1/13 (8)
Management of unsuccessful LA dilatation	*n* = 25
Hysterectomy	9 (36)
Withdrawal from screening	9 (36)
Dilatation under GA	5 (20)
Offered repeat smear only	1 (4)
LLETZ (GA)	1(4)

*Note*: All data is displayed as number (%) or median (interquartile range).

Abbreviations: GA, general anesthetic; LA, local anesthetic; LLETZ, large loop excision of the transformation zone.

^a^ Missing data (*n* = 6).

Of the 149 referrals for cervical dilatation, five patients opted for a general anesthetic without attempting the procedure under local anesthetic. Successful dilatation with LA was achieved in 119 (83%) patients. The median Hegar size used was 8 mm.^7^, ^8^ Six patients (4%) were unable to tolerate the speculum insertion and in 19 cases (13%), the procedure was unsuccessful.

Where LA dilatation was attempted, patients were asked whether they preferred a local versus a general anesthetic. The outpatient dilatation was reported as acceptable in 93% cases. Seven (5%) reported they would have preferred a general anesthetic and two (1%) were unsure. In 12 cases, a total of 15 complications were described. One patient experienced a vasovagal episode, which required no further management, and one patient experienced vaginal bleeding, fever, and back pain 10 days postprocedure which was managed in the community with oral co‐amoxiclav. There were a total of 13 reported episodes of restenosis (detected at 3 months postdilatation) in 12 patients. One patient was seen twice in the clinic; she initially underwent successful dilatation under LA, however, at follow‐up there was evidence of restenosis which was managed by with repeat dilatation under GA. Her second episode of restenosis was managed with a hysterectomy. Of the 13 episodes of restenosis, 8 (62%) patients were managed with hysterectomy, 2 patients underwent repeat dilatation under LA, 2 patients had repeat dilatation under GA, and 1 patient had a LLETZ under GA to rule out any preinvasive cervical pathology.

Of the unsuccessful procedures, nine (36%) were managed with hysterectomy, nine (36%) were withdrawn from the cervical screening program, five (20%) had a repeat dilatation under GA, one (4%) was managed with a LLETZ, and one (4%) (due to extensive comorbidities) was offered a repeat smear. Management was discussed at the local colposcopy multidisciplinary meeting for nine (36%) patients. In five cases the cervix was reported as flush with the vagina. In the remaining 14 failed procedures no specific details were documented.

Univariate analysis was conducted to determine which variables were associated with the degree of cervical stenosis and successful cervical dilatation under LA (Table 3). The number of previous LLETZ procedures (*p* = 0.004) and the subsequent Hegar dilatation achieved (*p* < 0.0001) were statistically associated with the degree of cervical stenosis. Patient age (*p* = 0.045) and the date of procedure (*p* = 0.003) were associated with procedure success; with a greater success rate in younger patients and chronological progression. No statistically significant variables were associated with episodes of restenosis.

**TABLE 3:** Univariate analysis

Factors affecting degree of stenosis	Severe stenosis	Complete stenosis	*p*‐Value
Median date of procedure	07 July, 2017	August 18, 2017	0.595
Age	48 (35, 54)	51 (40, 58)	0.13
Parity	2 (0, 3)	2 (0, 2)	0.469
Menopausal	30/74 (41)	30/63 (48)	0.487
No. previous LLETZ	1 (1,1)	1 (1, 2)	0.004
Previous O/G procedures	19/74 (26)	18/63 (29)	0.704
Presence medical comorbidities	19/74 (26)	22/63 (35)	0.239
Hegar dilatation achieved	8 (7, 8)	7 (0,8)	<0.0001
Restenosis	7/74 (9)	5/63 (8)	0.247
Factors affecting success of procedure	Successful LA Dilatation	Unsuccessful LA Dilatation	
Median date of procedure	August 18, 2017	June 09, 2016	0.003
Age	49 (36, 54)	53 (47, 61)	0.045
Parity	2 (0, 3)	1 (1, 2)	0.136
Menopausal	48/118 (41)	14/24 (58)	0.086
No. previous LLETZ	1 (1, 1)	1 (1, 2)	0.415
Previous O/G procedures	30/118 (25)	8/24 (33)	0.425
Presence of medical comorbidities	35/118	8/24 (33)	0.721

*Note*: All data is displayed as number (%) or median (interquartile range).

Abbreviations: GA, general anesthetic; LA, local anesthetic; LLETZ, large loop excision of the transformation zone; O/G, obstetric or gynecological.

### Cervical cytology, histology, and management

The cytological results from the first smear performed before and after dilatation (or hysterectomy) are reported in Table 4, alongside the subsequent management.

**TABLE 4:** Cytology, follow‐up, histology, and further management

	Predilatation	First postdilatation
Cytology result		
Normal	55 (37)	70 (47)
Normal, HPV positive	30 (20)	25 (17)
Borderline	5 (3)	4 (3)
Borderline, HPV positive	13 (9)	9 (6)
Low grade	10 (7)	9 (6)
Low grade, HPV positive	6 (4)	3 (2)
Moderate grade	1 (<1)	0 (0)
High grade	1 (<1)	0 (0)
Inadequate sample	3 (2)	2 (1)
No result	24 (16)	26 (18)
Follow‐up smear management		
Discharged to GP (Routine)		48 (32)
Repeat Colp clinic, discharged to GP (Routine)		22 (15)
Repeat Colp clinic, discharged to GP (annual/ 6mo)		11 (7)
Repeat Colp clinic, on‐going hospital surveillance		30 (20)
Repeat Colp clinic, discharged (no further smears)		4 (3)
Did not attend		11 (7)
No follow‐up required		11 (7)
Missing data		3 (2)
Patients requiring LLETZ		8
Adequate colposcopy		20/48 (42)
Histology	From LLETZ	From Hysterectomy
HPV/benign/inflammatory	7 (78)	19 (90)
Grade 1 ectocervical adenocarcinoma, (FIGO Stage 1A1)	0	1 (5)
CIN1	2 (22)	0
CIN3	0	1 (5)

*Note*: All data is displayed as number (%).

Abbreviations: CIN, cervical intraepithelial neoplasia; Colp, colposcopy; FIGO, The International Federation of Gynecology and Obstetrics; GP, general practice; HPV, human papilloma virus; LLETZ, large loop excision of transformation zone.

On comparison with predilatation cytology, there was an increase in the number of normal results and an overall decrease in the detection of HPV. This difference is likely to represent the transient nature of HPV infection, plus women who have undergone cervical treatment. Predilatation, one patient had high‐grade dyskaryosis, she has previously had one incomplete LETTZ and was offered a hysterectomy.

In terms of postdilatation management, 70 (47%) women had normal cytology, with 48 (32%) returning to the national cervical screening program under the care of their general practitioner. Sixty‐four women were followed up with repeat hospital colposcopy clinics; 22 (15%) were subsequently discharged to their GP for routine cervical screening, and 11 (7%) were discharged with more frequent smears. Four (3%) patients who had hysterectomy required no further screening after normal vault cytology. In 30 (20%) patients, active hospital surveillance was required. Colposcopy examinations were described in 48 patients, 20 (42%) of which were adequate for assessing the cervix. Of these patients, eight required LLETZ and two patients opted for hysterectomy. In addition, six further hysterectomies were performed for patient choice (*n* = 2), menorrhagia (*n* = 2), endometriosis (*n* = 1), and pelvic organ prolapse (*n* = 1) (Data S1, Supporting Information).

A total of 10 patients with cervical stenosis underwent LLETZ; 1 patient after failed dilatation, 1 patient with restenosis, and 8 patients postdilatation. The patient who underwent unsuccessful dilatation was managed with LLETZ under GA. She had one previous LLETZ and had completely fused, severe stenosis with vaginal flush. The patient with restenosis had a LLETZ (under GA) to rule out any preinvasive cervical pathology. She had not previously undergone LLETZ and was offered a choice of attempting a repeat smear, withdrawal from screening, LLETZ, or hysterectomy. Table 5 displays the characteristics of the eight patients who required LLETZ at their next postdilatation assessment. The LLETZ were performed for diagnostic rather than therapeutic purposes to rule out malignant and preinvasive disease following abnormal smear results after cervical dilatation. Five of eight had inadequate colposcopy and underwent LLETZ to rule out any preinvasive cervical pathology. The remaining three patients had CIN changes on colposcopy; one of which (with CIN3) had an incomplete LLETZ and had a hysterectomy.

**TABLE 5:** Reasons for LLETZ after successful cervical dilatation

Pt	Previous LLETZ	Stenosis	Successful LA dilatation	Preresult	Postresult	Colposcopy	Histology	Comment
1	2	Complete	y	Borderline + HPV	Borderline + HPV	Inadequate	HPV/benign/inflammatory	Repeat smear 1 year after LLETZ
2	1	Complete	y	NAD + HPV	NAD + HPV	Inadequate	HPV/benign/inflammatory	Repeat smear in 6 months by GP after LLETZ
3	1	Significant	Required GA	Borderline	Borderline	Inadequate	HPV/benign/inflammatory	Repeat smear in 6 months by GP after LLETZ
4	1	Significant	y	Borderline	Borderline	Inadequate ×2	HPV/benign/inflammatory	Initial smear successful (3 months), inadequate colp, restenosis on repeat 3mo smear (second LA dilatation), successful smear (3 months), inadequate colp, LLETZ performed, repeat smear in 1 year
5	1	Complete	y	Borderline	NAD + HPV	Inadequate	HPV/benign/inflammatory	Repeat smear in 6 months by GP after LLETZ
6	1	Complete	y	Low grade	Low grade	LLETZ indicated	CIN1	Repeat smear in 6 months by GP after LLETZ
7	0	Significant	y	Low grade	Low grade	LLETZ indicated	CIN1	Repeat smear in 1 year by GP after LLETZ
8	1	Significant	y	NAD + HPV	NAD + HPV	LLETZ indicated	CIN3	Incomplete excision of CIN3, patient had hysterectomy, vault smear in 6 months

Abbreviations: CIN, cervical intraepithelial neoplasia; Colp, colposcopy; GA, general anesthetic; GP, general practice; HPV, human papilloma virus; LA, local anesthetic; LLETZ, large loop excision of transformation zone; NAD, no abnormality detected.

Histological results from LLETZ showed seven (78%) benign samples and two (22%) cases of CIN1. From hysterectomy there were 19 (90%) benign samples, 1 (5%) CIN3, and 1 (5%) grade 1 ectocervical adenocarcinoma, (FIGO Stage 1A1). The latter patient was followed up 3 months postdilatation with borderline endocervical changes and HPV positive. She had previously had a LLETZ for high‐grade dyskaryosis. Her colposcopy examination was described as unremarkable and her case was discussed at the colposcopy multidisciplinary meeting. She was subsequently offered either a LLETZ or hysterectomy.

## Discussion

This is the largest prospective case series of patients undergoing mechanical dilatation of the cervix under LA in the outpatient setting.

In our study, 79% of women had previously undergone a LLETZ procedure. The presence of cervical stenosis in women who have received cervical treatments is well documented.^2^, ^4^, ^5^, ^6^, ^7^, ^8^, ^9^ Statistical associations with cervical stenosis have been described including LLETZ procedures^4^, ^5^ (particularly Top‐hat LLETZ^23^), the volume,^5^ or height^2^ of excised cervical tissue and lesions confined to the endocervix.^2^ We identified a statistically significant relationship between the severity of stenosis and the number of previous LLETZ procedures (*p* = 0.004) as reported in the literature.

The development of cervical stenosis has also been associated with advancing age^8^ and the menopause.^3^ Penna et al. reported a greater incidence of cervical stenosis in the postmenopausal population, however, the risk was reduced with estrogen use.^24^ Contrary to the literature, a postmenopausal status was only reported in 42% of women referred to our clinic and there was no statistical association between age and menopausal status with the severity of stenosis. This might be the result of referrals received directly from colposcopy clinic following LLETZ in women from the cervical screening group.

One hundred forty‐eight women were referred to our clinic with cervical stenosis, 1 of which underwent two LA dilatations. Success rates were high (83%), with the vast majority (93%) retrospectively stating preference for an outpatient dilatation. Similarly, Bettocchi et al. reported successful hysteroscopic management of cervical stenosis in the outpatient setting in 98.5% of cases using hysteroscopy maneuvers, miniature mechanical instruments, or bipolar diathermy.^3^ Of the 30 000 women attending for outpatient hysteroscopy, 4.3% were unable to tolerate the procedure.^3^ We observed a similar rate among our cohort. Successful outpatient dilatation was also reported in randomized control trial conducted by Lin et al. (*n* = 450). Postmenopausal women who had recently undergone LLETZ procedures were randomized to receive regular postprocedural Hegar dilatation versus no or standardized care. The control group did not undergo any intervention measures of cervical dilatation, but only observed the wound surface and recorded the complications regularly every month after LLETZ. The prophylactic approach resulted in significantly few cases of cervical stenosis when compared to controls at 6 months, with no major complications. The authors recommend regular rigid dilatation for the first 2 months post‐LLETZ to be conducted in the outpatient setting.^25^ Although this approach did safely reduce cervical stenosis post‐LLETZ, offering all patients monthly rigid dilatation may not be practically feasible. This is especially the case as it is recommended that the procedure is performed by a senior clinician to maximize success rates and minimize risk of complications.

In 19 cases (13%), cervical dilatation under LA was unsuccessful, however, over the 5‐year period, the success rate significantly improved (*p* = 0.03). Procedure success was also statistically influenced by younger age (*p* = 0.045) and the degree of stenosis; with greater Hegar dilatation achieved in those with severe rather than complete stenosis (*p* < 0.0001). From our experience, success rate is based on appropriate case selection and also increasing experience of an operator. In view of a small number of cases needed cervical dilation, the procedure should be performed by an experience operator or a lead clinician.

Twenty‐five patients underwent hysterectomies where 9 patients had a failed procedure under LA, and 8 patients experienced restenosis. In our cohort, histology from hysterectomy was reported as 90% benign, with one case of CIN3 and one grade 1 adenocarcinoma (FIGO Stage 1A1). Newman and Finan (2003) retrospectively reviewed 25 women with cervical stenosis who had undergone hysterectomy. The follow‐up histology demonstrated 64% benign pathology, 12% cervical dysplasia, and 4% incidence of uterine cancer, with the authors concluding that where cytology was not possible, hysterectomy was a reasonable option.^26^

According to the NHS cervical screening program (NHSCSP) management guidelines, colposcopy MDMs should be incorporated into patient care, and may be useful when managing patients with cervical stenosis and that cervical dilatation is considered for all patients with cervical stenosis. Only a third of patients with unsuccessful cervical dilated, management was discussed at the MDM for further management. This pathway has streamlined management of cervical stenosis and avoided premature discussion of such cases in the MDM. The outcome from MDM would usually be consideration for a hysterectomy in the presence of high‐grade dyskayosis or cervical glandular intraepithelial neoplasia (CGIN). If neither hysterectomy nor dilatation is deemed appropriate, a joint decision could be made with the patient to be withdrawn from the national screening program.

There were no reported complications in our case series in women who had successful cervical dilatation. There has been discussion into the safety of rigid cervical dilatation, with reports of cervical tears, uterine perforation and the creation of a false cervical passage.^11^, ^27^ Arsenijevic et al. reported greater cervical tissue damage using Hegar dilators versus balloon dilatation.^15^ The use of prostaglandins to pharmacologically prime the cervix has also been described to reduce the risk of laceration by decreasing the instrumental force required.^28^, ^29^ Given that common adverse effects include preprocedure pain and bleeding, we do not recommend routine use of medical agents.^28^, ^29^ Furthermore, in a 5‐year follow‐up study, the majority of women undergoing medical dilatation required further cervical treatment, with the authors concluding that modern excisional surgery is likely more efficient.^30^

Restenosis of the cervix occurred in 11% of women who successfully underwent dilatation in our clinic. In univariate analysis, no statistically significant risk factors were identified. The rate of restenosis is comparable to study by Valle et al. reported.^31^ The rate of restenosis is also comparable to study by Valle et al. where cervical dilatation was performed under GA. With similar restenosis rate, procedure undertaken under LA would be a safer and a more cost‐effective approach without compromising on restenosis rate.

In England, the NHSCSP is estimated to prevent 70% of cervical cancer deaths, which could be increased to 83% with improved compliance.^32^ In our study, highly successful dilatation has allowed women to undergo cervical screening by obtaining adequate smear. In a third of patients, normal cytology on their first follow‐up smear resulted in being discharged to their GP to return to three or 5 yearly screening. Further hospital follow‐up was required in a quarter of patients before they were discharged, with 17% requiring ongoing surveillance in secondary care. Ten patients went on to have treatment, eight LLETZ procedures, and two requiring hysterectomies.

Of the 48 patients requiring colposcopy in their postdilatation follow‐up, an adequate assessment was only possible in 42%. A prospective cohort study reported inadequate colposcopies in 14% patients post‐LLETZ, which was significantly associated with a history of previous LLETZ procedure and thicker tissue excision.^4^Successful cervical dilatation has made adequate colposcopy in almost half of these cases and avoided unnecessary further intervention.

The majority of the histological results were benign, however, cervical intraepithelial neoplasia (CIN 1) and CIN3 were detected in three patients, and one patient undergoing hysterectomy was found to have a grade 1 ectocervical adenocarcinoma. Considering the majority of women presenting with cervical stenosis have had previous excisional therapy for high‐grade CIN, it is essential that dilatation is considered to avoid underdiagnosed preinvasive cervical disease with vigorous surveillance recommended. Successful cervical dilatation also enable patient to maintain adequate smear within the national cervical screening program to fulfill the successful aim of cervical screening in preventing cervical cancer.

In conclusion, we achieved good success rates in treated cervical stenosis with Hegar dilatation under LA. Dilatation was performed safely in all patients with no major adverse events. We recommend careful case selection and dilatation to be performed by experienced clinician to minimize complications and maximize success rates. For the vast majority of women, the procedure was well tolerated and preferred to using GA. Given that stenosis commonly occurs in women previously treated with cervical excisions for high‐grade CIN, it is vital that women continue with their regular smear surveillance.

Rigid cervical dilatation in the outpatient setting provides a safe, effective, and instantaneous treatment for women who have otherwise inadequate cytology or colposcopic assessment. However, given that 1 in 10 women experienced restenosis, patients should be counseled about the possibility of requiring further management.

## Conflict of interest

Kirsty V. Biggs, Mallikarjun Kodampur, and San S. Hoo disclose no conflicts of interest.

## Supporting information


**Data S1** Indications for hysterectomy

## Data Availability

Data available on request from the authors

## References

[1] Baldauf JJ , Dreyfus M , Wertz JP , Cuénin C , Ritter J , Philippe E . Consequences and treatment of cervical stenoses after laser conization or loop electrosurgical excision. J Gynecol Obstet Biol Reprod (Paris). 1997;26(1):64–70. 9091546

[2] Baldauf JJ , Dreyfus M , Ritter J , Meyer P , Philippe E . Risk of cervical stenosis after large loop excision or laser conization. Obstet Gynecol. 1996 Dec;88(6):933–8. 8942830 10.1016/S0029-7844(96)00331-6

[3] Bettocchi S , Bramante S , Bifulco G , Spinelli M , Ceci O , Fascilla FD , et al. Challenging the cervix: strategies to overcome the anatomic impediments to hysteroscopy: analysis of 31,052 office hysteroscopies. Fertil Steril. 2016;105(5):e16–7. 26873675 10.1016/j.fertnstert.2016.01.030

[4] Carcopino X , Mancini J , Gondry J , Chevreau J , Lamblin G , Atallah A , et al. Risk factors of inadequate colposcopy after large loop excision of the transformation zone: a prospective cohort study. J Low Genit Tract Dis. 2018;22(1):31–7. 29271854 10.1097/LGT.0000000000000357

[5] Suh‐Burgmann EJ , Whall‐Strojwas D , Chang Y , Hundley D , Goodman A . Risk factors for cervical stenosis after loop electrocautery excision procedure. Obstet Gynecol. 2000;96(5 Pt 1):657–60. 11042296

[6] Wright TC , Gagnon S , Richart RM , Ferenczy A . Treatment of cervical intraepithelial neoplasia using the loop electrosurgical excision procedure. Obstet Gynecol. 1992;79(2):173–8. 1731281

[7] Hallam NF , West J , Harper C , Edwards A , Hope S , Merriman H , et al. Large loop excision of the transformation zone (LLETZ) as an alternative to both local ablative and cone biopsy treatment: a series of 1000 patients. J Gynecol Surg. 1993;9(2):77–82. 10146250 10.1089/gyn.1993.9.77

[8] Houlard S , Perrotin F , Fourquet F , Marret H , Lansac J , Body G . Risk factors for cervical stenosis after laser cone biopsy. Eur J Obstet Gynecol Reprod Biol. 2002;104(2):144–7. 12206927 10.1016/s0301-2115(02)00062-3

[9] Keijser KG , Kenemans P , van der Zanden PH , Schijf CP , Vooijs GP , Rolland R . Diathermy loop excision in the management of cervical intraepithelial neoplasia: diagnosis and treatment in one procedure. Am J Obstet Gynecol. 1992;166(4):1281–7. 1566785 10.1016/s0002-9378(11)90622-x

[10] Pschera H , Kjaeldgaard A . Haematocervix after conization diagnosed by ultrasonography. Gynecol Obstet Investig. 1990;29(4):309–10. 2193859 10.1159/000293343

[11] Bradley LD . Complications in hysteroscopy: prevention, treatment and legal risk. Curr Opin Obstet Gynecol. 2002;14(4):409–15. 12151831 10.1097/00001703-200208000-00008

[12] Kosinska‐Kaczynska K , Ciechanowicz P , Saletra A , Szymusik I , Wielgos M . Two methods of cervix ripening: intracervical Foley catether and dinoprostone ‐ which one is actually more efficient? Neuro Endocrinol Lett. 2015;36(3):257–61. 26313393

[13] Gelber S , Sciscione A . Mechanical methods of cervical ripening and labor induction. Clin Obstet Gynecol. 2006;49(3):642–57. 16885669 10.1097/00003081-200609000-00022

[14] Biron‐Shental T , Fishman A , Fejgin MD . Medical and mechanical methods for cervical ripening. Int J Gynaecol Obstet. 2004;85(2):159–60. 15099778 10.1016/j.ijgo.2003.08.006

[15] Arsenijevic S , Vukcevic‐Globarevic G , Volarevic V , Macuzic I , Todorovic P , Tanaskovic I , et al. Continuous controllable balloon dilation: a novel approach for cervix dilation. Trials. 2012;22(13):196. 10.1186/1745-6215-13-196 PMC3543240 23088906

[16] Shindo R , Aoki S , Yonemoto N , Yamamoto Y , Kasai J , Kasai M , et al. Hygroscopic dilators vs balloon catheter ripening of the cervix for induction of labor in nulliparous women at term: retrospective study. PLoS One. 2017;12(12):e0189665. 29272277 10.1371/journal.pone.0189665 PMC5741218

[17] Jozwiak M , Oude Rengerink K , Benthem M , van Beek E , Dijksterhuis MGK , de Graaf IM , et al. Foley catheter versus vaginal prostaglandin E2 gel for induction of labour at term (PROBAAT trial): an open‐label, randomised controlled trial. Lancet. 2011;378(9809):2095–103. 22030144 10.1016/S0140-6736(11)61484-0

[18] Hadadian S , Fallahian M . Assessing the efficacy of vaginal hyoscine butyl bromide on cervical ripening prior to intrauterine procedures: a double‐blinded clinical trial. Int J Reprod Biomed. 2016;14(11):709–12. 27981257 PMC5153577

[19] Hadadianpour S , Tavana S , Tavana A , Fallahian M . Immediate dilation of a tight or stenotic cervix by intra‐procedural administration of hyoscine butylbromide: a clinical trial. Int J Reprod Biomed. 2019;17(4):253–60. 10.18502/ijrm.v17i4.4550 PMC6686652 31435605

[20] Koyama S , Kobayashi M , Tanaka Y , Kubota S , Nakamura R , Isobe M , et al. Complete cervical stenosis after conization: timing for the minimally invasive reconstructive surgery. GMIT. 2014;3(2):57–60.

[21] Li X , Li J , Wu X . Incidence, risk factors and treatment of cervical stenosis after radical trachelectomy: a systematic review. Eur J Cancer. 2015;51(13):1751–9. 26049687 10.1016/j.ejca.2015.05.012

[22] Mathew M , Mohan AK . Recurrent cervical stenosis ‐ a troublesome clinical entity. Oman Med J. 2008;23(3):195–6. 22359714 PMC3282328

[23] Mossa MA , Carter PG , Abdu S , Young MPA , Thomas VA , Barton DPJ . A comparative study of two methods of large loop excision of the transformation zone. BJOG. 2005;112(4):490–4. 15777450 10.1111/j.1471-0528.2005.00427.x

[24] Penna C , Fambrini M , Fallani MG , Pieralli A , Scarselli G , Marchionni M . Laser CO2 conization in postmenopausal age: risk of cervical stenosis and unsatisfactory follow‐up. Gynecol Oncol. 2005;96(3):771–5. 15721425 10.1016/j.ygyno.2004.11.012

[25] Lin J , Meng Y , Chen Y , Li Z , Xu Y , Wu D . A new approach to prevent cervical stenosis in postmenopausal women after loop electrosurgical excision procedure: a randomized controlled trial. Sci Rep. 2020;10(1):8512. 32444670 10.1038/s41598-020-65170-2 PMC7244737

[26] Newman C , Finan MA . Hysterectomy in women with cervical stenosis. Surgical indications and pathology. The. J Reprod Med. 2003;48(9):672–6. 14562629

[27] Cheng MC , Karim SM , Ratnam SS . Preoperative cervical dilatation with 15 (S) 15 methyl prostaglandin E2 methyl ester in first trimester nulliparae. A double blind study. Contraception. 1975;12(1):59–67. 1095297 10.1016/s0010-7824(75)80037-0

[28] Ponce G , de León R , Wing DA . Misoprostol for termination of pregnancy with intrauterine fetal demise in the second and third trimester of pregnancy ‐ a systematic review. Contraception. 2009;79(4):259–71. 19272495 10.1016/j.contraception.2008.10.009

[29] Fiala C , Gemzell‐Danielsson K , Tang OS , von Hertzen H . Cervical priming with misoprostol prior to transcervical procedures. Int J Gynaecol Obstet. 2007;99(Suppl 2):S168–71. 17961571 10.1016/j.ijgo.2007.09.005

[30] Johnson N , Brady J . Dilating the cervix medically to overcome an unsatisfactory colposcopy: 5 year follow up. Eur J Obstet Gynecol Reprod Biol. 1996;69(2):125–7. 8902445 10.1016/0301-2115(95)02512-x

[31] Valle RF , Sankpal R , Marlow JL , Cohen L . Cervical stenosis: a challenging clinical entity. J Gynecol Surg. 2002;18(4):129–43.

[32] Landy R , Pesola F , Castañón A , Sasieni P . Impact of cervical screening on cervical cancer mortality: estimation using stage‐specific results from a nested case‐control study. Br J Cancer. 2016;115(9):1140–6. 27632376 10.1038/bjc.2016.290 PMC5117785

