# Supplementary material for: Mechanical dilatation of the stenosed cervix under local anesthesia: A prospective case series
Source: J Obstet Gynaecol Res. 2022 Feb 7;48(4):956–65. doi: 10.1111/jog.15179 (PMC9303640; doi:10.1111/jog.15179)
Supplement: Supplementary file 1 — Data S1 Indications for hysterectomy [file JOG-48-956-s001.docx]

| **Supplementary material 1: Indications for hysterectomy** | **n=25** |
| --- | --- |
| Failed procedure under local anaesthetic | 9 |
| Restenosis | 8 |
| Cervical treatment | 2 |
| Heavy menstrual bleeding | 2 |
| Patient choice | 2 |
| Endometriosis | 1 |
| Pelvic organ prolapse | 1 |
